# MAPI–MoS_2_ quantum dot composite films as active layers for efficient photovoltaics†

**DOI:** 10.1039/d5na00485c

**Published:** 2025-06-23

**Authors:** Subha Sadhu, Ankur Kambley, Talitha R. C. Santos, Abhijit Ganguly, Slavia Deeksha Dsouza, Dilli Babu Padmanaban, Pagona Papakonstantinou, Paul Maguire, Vladimir Svrcek, Davide Mariotti

**Affiliations:** a Department of Chemistry, Institute of Science, Banaras Hindu University Varanasi India; b Renewable Energy Advanced Research Center, National Institute of Advanced Industrial Science and Technology (AIST) Tsukuba Japan; c Group of Nanostructured Devices, Federal University of Paraná (Physics Department) Curitiba PR Brazil; d School of Engineering, Ulster University Belfast UK; e School of Physics & Astronomy, University of Glasgow Glasgow UK; f Department of Design, Manufacturing & Engineering Management, University of Strathclyde Glasgow UK davide.mariotti@strath.ac.uk

## Abstract

In this work we have incorporated MoS_2_ quantum dots having outstanding optoelectronic properties with methyl ammonium lead iodide (MAPI) to form a composite absorber material for photovoltaic applications. The inclusion of MoS_2_ quantum dots in the perovskite layer improves the absorption and charge transport properties of the active layer, in part due to the quantum dots contributing to defect passivation at the MAPI grain interfaces. The photocurrent density increases when the MoS_2_ quantum dots are introduced in the device structure, resulting in efficiency improvements of 14% and 28% for devices fabricated in different laboratories.

## Abstract 2

In this work we have incorporated MoS_2_ quantum dots having outstanding optoelectronic properties with methyl ammonium lead iodide (MAPI) to form a composite absorber material for photovoltaic applications.

## Graphical abstract

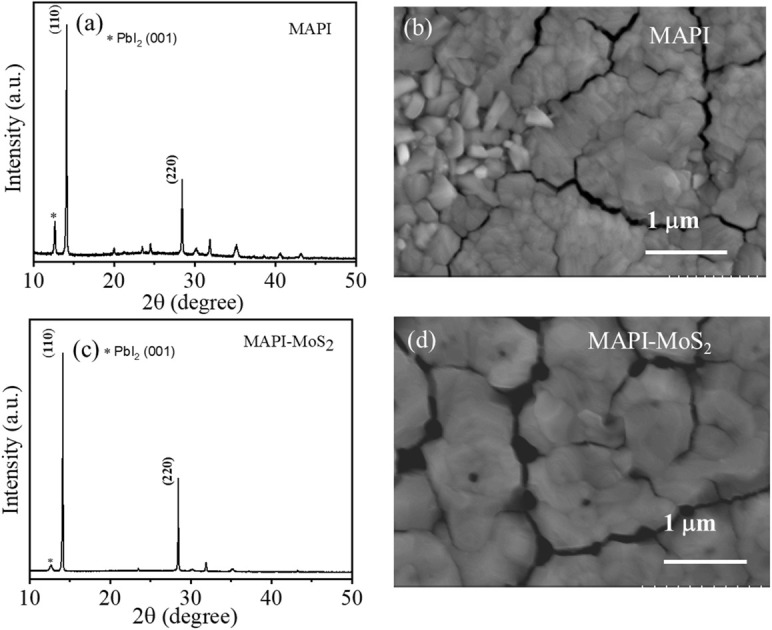

## Introduction

Hybrid organic–inorganic perovskites exhibit intriguing optoelectronic properties that have sparked great interest for photovoltaics and other applications.^1–12^ The high absorption cross section in the visible range, high charge carrier mobility and low fabrication cost are the main reasons for the growing worldwide interest in halide perovskite research. Among all the perovskites, methylammonium lead iodide (CH_3_NH_3_PbI_3_), or MAPI, is one of the most investigated halides, as within just five years of research the power conversion efficiency (PCE) of MAPI-based devices increased drastically.^1^ Until now the highest PCE for single junction perovskite solar cell is above 26% whereas for perovskite tandem solar cells, the efficiency is >30%, which surpasses the maximum theoretical PCE limit of 29.3% for single junction silicon solar cells.^13–17^ The tetragonal CH_3_NH_3_PbI_3_ ionic crystal structure consists of an inorganic part (PbI_3_)^−^ and an organic part (CH_3_NH_3_)^+^. The Pb and I atoms are arranged in an octahedral configuration PbI_6_, whereas the methylammonium cations CH_3_NH_3_^+^ fill the intermediate free spaces between the octahedra, providing charge equilibrium. Like other ionic crystals, MAPI possesses under-coordinated ions and dangling bonds at the surface, causing defects and trap sites and often negatively impacting the photovoltaic performance. Thus, passivation of perovskite interfaces is an avenue often pursued to improve photovoltaic performance, resulting from increased charge carrier mobility and decreased recombination.

MoS_2_ crystals possess a 2-dimensional (2D) layered structure and a few layers of MoS_2_ have shown excellent optoelectronic properties with absorption coefficients even higher than those of MAPI.^18^ MoS_2_ and other 2D materials have been used to increase charge transfer properties and attain better energy level alignment in perovskite solar cells.^19^ However, the inclusion of 2D materials was generally aimed at enhancing carrier extraction rather than enhancing absorption and carrier generation, requiring devices with different architectures. Furthermore, there has been no attempt to use quantum confined MoS_2_ in MAPI. In our previous work, we used MoS_2_ quantum dots (QDs), the 0-dimensional form of MoS_2_, within a formamidinium lead iodide (FAPI) absorber. We have shown that through a type-I alignment between FAPI and the QDs, it was possible to increase carrier generation and the overall performance of the photovoltaic devices.^20,21^ In this contribution, we have investigated the viability of MoS_2_ QDs within a MAPI absorber, to verify their alignment as well as the structural stability and opto-electronic performance of the corresponding MAPI–MoS_2_ QD composite film. Because of the different alignment with MAPI, compared to FAPI, substantial different outcomes can be expected. We also expect the QDs to passivate the MAPI surface as Pb^2+^ has a strong interaction with S^2−^.^22^ Thus, inclusion of MoS_2_ in the MAPI layer is likely to increase absorption, which can contribute to carrier generation, as well as reduce dangling bonds and trap sites through the interaction of Pb–S coordination bonding. It is important to note that in this type of architecture, MoS_2_ QDs are dispersed in the perovskite absorber, forming a composite MAPI–MoS_2_ QD absorber layer, and there is no attempt to re-produce the more established bulk-heterojunction architecture. Ideally, we expect both positive and negative carriers generated in the QDs to be both transferred to and transported through the perovskite. However, this approach relies on an appropriate alignment of the energy levels.^21^

We fabricated thin films of MAPI–MoS_2_ QDs with different amounts of QDs and optimized the concentration for maximum absorption of the active layer. We carried out a detailed structural and morphological characterization study of the thin film. The inclusion of MoS_2_ QDs in the MAPI layer was confirmed from the analysis of Raman and X-ray photoelectron spectra. We analysed the band energy diagram of the corresponding materials and then produced device test structures at two different laboratories, following the same fabrication recipe. The results confirmed consistent improvement in the photoconversion efficiency, mainly driven by higher photocurrent for all the devices with the MoS_2_ QDs.

## Experimental method

### Synthesis of MoS_2_ quantum dots

MoS_2_ QDs were synthesized by grinding high-purity bulk MoS_2_ powder (<2 μm, 99.0%, Sigma-Aldrich) *via* an ionic liquid-assisted mechanical exfoliation method, followed by size selection through sequential centrifugation steps, as reported in our earlier publication.^23^ Briefly, the process involved the mechanical grinding of MoS_2_ platelets in an adequate quantity of room-temperature ionic liquid (RTIL, 1-butyl-3-methylimidazolium hexafluorophosphate, ≥97.0%, Sigma-Aldrich), using an agate mortar and pestle grinder system (RM200, Retsch GmbH). During grinding, the RTIL protected every newly exposed MoS_2_ surface by adsorbing onto the surface, keeping the QDs separated and avoiding restacking. After grinding for a sufficiently long duration, the resulting gel was subjected to multiple washing steps in a mixture of acetone (≥99.8%) and *N*,*N*-dimethylformamide (DMF, ≥99.9%), with a gradually increasing proportion of DMF to remove the remaining RTIL using centrifugation at 10 000 rpm. Finally, the clean ground product, consisting of an assortment of QDs of various sizes and thicknesses, was dispersed homogeneously in pure DMF and subsequently subjected to sequential centrifugation steps with increasing centrifugation speeds from 500 rpm to 10 000 rpm using a Thermo Scientific Sorvall ST-16 centrifuge system. The sequential centrifugation of the supernatant at progressively higher centrifugation speeds for longer durations allowed the isolation of thinner and smaller QDs. This study chose the thinnest and smallest QDs, pelleted after centrifugation at 10 000 rpm. The lateral size of the QDs is ∼20 nm with an average thickness of up to 7 layers (∼4 nm).

### Fabrication of films made of MAPI and MAPI with MoS_2_ QDs (MAPI–MoS_2_)

MAPI films were deposited on In-doped SnO_2_ (ITO) coated glass substrates by spin coating a precursor solution, which was prepared by dissolving methylammonium lead iodide powder (1 M) in dimethyl sulfoxide (DMSO) and DMF solvent (volume ratio 1 : 4). To incorporate the MoS_2_ QDs in the precursor solution, MoS_2_ QDs at appropriate concentrations (0.2–0.6 mg mL^−1^) were dispersed in DMF and sonicated for 1 h and then added to the methylammonium lead iodide powder with DMSO in a volume ratio of 4 : 1. All the preparation steps were carried out under ambient conditions.

### Device fabrication

The perovskite solar cells were fabricated following slightly modified previously reported methods.^21,24–26^ The ITO substrates were sonicated in Hellmanex soap solution, isopropanol and then acetone for 10 min each and then treated with an oxygen plasma for 15 min. To deposit the TiO_2_ blocking layer, 0.5 mL of 0.15 M titanium diisopropoxide dis(acetylacetonate) (Sigma-Aldrich, 75 wt% in isopropanol) in 1-butanol (Sigma-Aldrich, 99.8%) was spin-coated on the cleaned ITO substrate at 700 rpm for 8 s, 1000 rpm for 10 s, 2000 rpm for 40 s and annealed at 400 °C for 1 h. Thereafter, the mesoporous TiO_2_ layer was deposited above the TiO_2_ blocking layer by spin-coating 1 mL of 30NR TiO_2_ paste (Dyesol) at 2000 rpm for 20 s, followed by annealing at 400 °C for 2 h. After that, 50 μL of the precursor solution was spin-coated on the substrate at 4000 rpm for 30 s; after 20 s of spinning, 0.5 mL of diethyl ether was added drop-wise onto the spinning substrate for 5 s to fabricate a transparent film. The substrate was then heated at 100 °C for 10 min. To deposit the hole transport layer, 30 μL of spiro-OMETAD, 20 μL of tertiary butyl pyridine and 17.5 μL of lithium bis(trifluoromethanesulfonyl)imide (Li-TFSI) solution, (520 mg Li-TSFI in 1 mL acetonitrile (Sigma-Aldrich, 99.8%)), were added in 1 mL of chlorobenzene solution. This solution was spin-coated on the perovskite layer at 3000 rpm for 30 s. The substrate was kept overnight in a vacuum and thereafter, 100 nm of an Au layer, forming a counter electrode, was deposited by magnetron sputtering using argon plasma (Zepto Plasma Cleaner, Diener) at a constant current of 0.15 A for 15 min with a working pressure of 1.5 × 10^−2^ mbar. Batches of devices were produced at two different laboratories by different researchers (B1-devices and B2-devices). Each substrate contained 2 (B1-devices) or 6 (B2-devices) devices. All fabrication steps were carried out under ambient conditions for B1-devices while these were carried out in a glovebox with humidity <1% for B2-devices. Furthermore B1-devices had an active area of 4 mm^2^ while B2-devices were produced with a 25 mm^2^ active area. In both sets of devices, we observed that the fabrication process would require improvement to ensure uniform distribution of the MoS_2_ QDs across the substrate's surface area. This introduced variations in devices at the edges of the substrate surface area, which we have excluded from our analysis. In particular we believe that spin-coating would be better replaced with other methodologies such as spray coating or slot-die coating.

### Materials characterization

The crystalline phase of MAPI was confirmed by powder X-ray diffraction using a PANalytical X’PERT PRO instrument and iron-filtered Cu-K_α_ radiation (*λ* = 1.5406 Å) in the 2*θ* range of 10–50^°^ with a step size of 0.02^°^. An FEI Quanta 200 scanning electron microscope (SEM) was used to study the morphology of the films. The chemical composition and oxidation states were studied by X-ray photoelectron spectroscopy (XPS). Moreover, to determine the valence band maximum and Fermi level of the thin films, ultraviolet photoelectron spectroscopy (UPS) was performed, using an ESCALAB 250 Xi microprobe spectrometer (Thermo Fisher Scientific, UK) equipped with an Al-X-ray source and He(i) and He(ii) ultraviolet (UV) sources. The chamber pressure was 10^−9^ bar, and the spot size was 400 μm. Optical properties were investigated using an ultraviolet-visible (UV-vis) spectrometer (PerkinElmer Lambda 650S) equipped with an integrating sphere. A Kelvin probe from KP technology, equipped with an air photoemission spectroscopy (APS) module, was used to determine the valence band edge by measuring the photoemission of electrons from the sample. APS measurements were performed by using a deuterium lamp source and surface photovoltage was measured using a monochromatic white light source. Current density–voltage characteristics were calculated by irradiating the cell with 100 mW cm^−2^ light (450 W xenon lamp, Oriel instrument). A 1 sun AM 1.5 G filter was used to simulate the solar spectrum. The active area of the cell was 1 mm^2^. The photocurrent was measured by using a Keithley 2400 source measure unit.

## Results and discussion

**Fig. 1:**
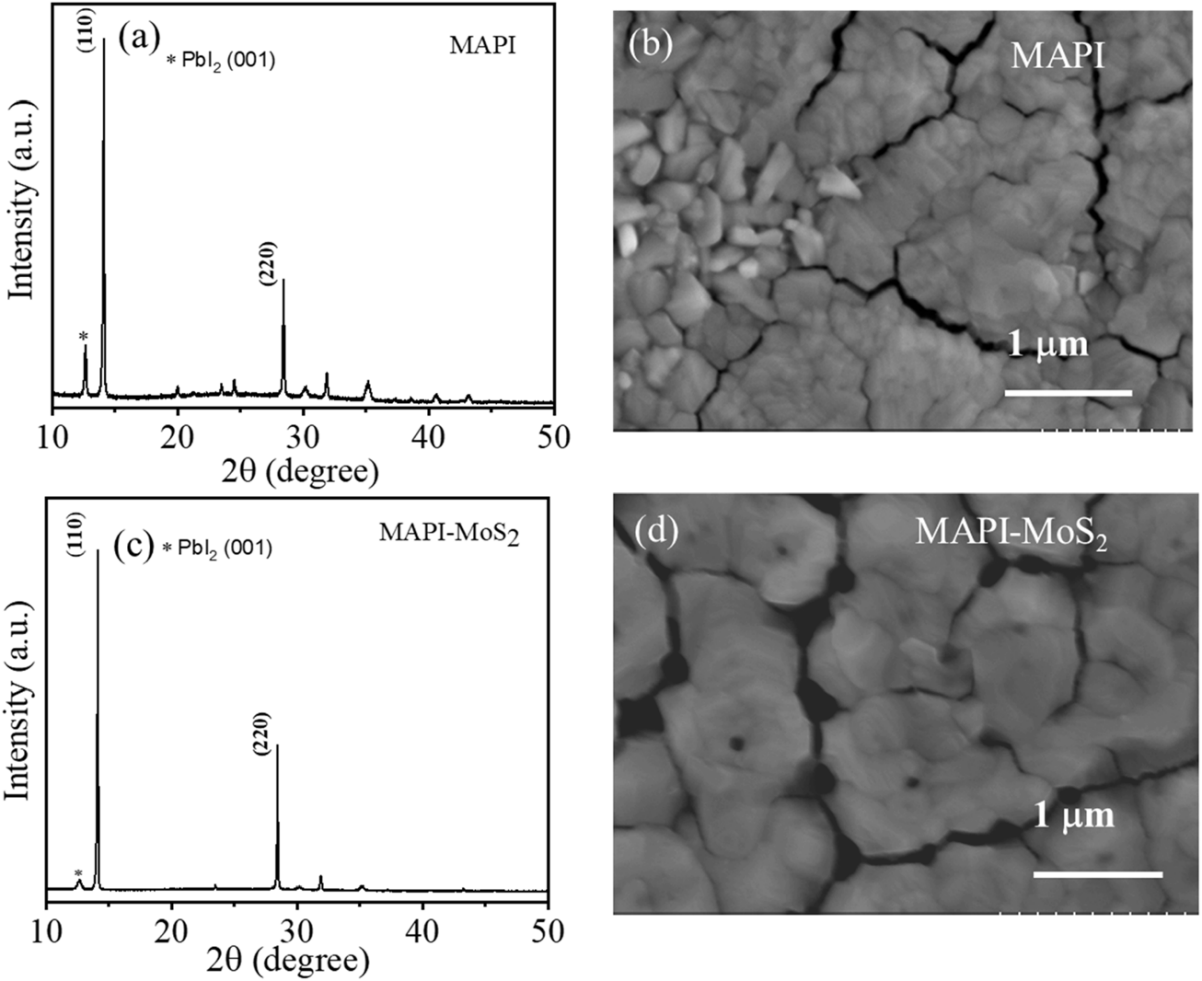
XRD patterns of (a) MAPI and (c) MAPI–MoS_2_ films and SEM images of (b) MAPI and (d) MAPI–MoS_2_ films on the ITO substrate at 0.4 mg mL^−1^ MoS_2_ QD concentration.

Thin films of MAPI and MAPI–MoS_2_ at different QDs concentrations (0.2 mg mL^−1^, 0.4 mg mL^−1^ and 0.6 mg mL^−1^) were fabricated and both SEM and XRD analyses were carried out (see the ESI, Fig. S1, S2 and Table S1†). Qualitatively, we observed differences between the SEM images of the MAPI film and those of the films with QDs, with a morphology suggesting smaller grains at the surface when the QDs were introduced (Fig. 1b and d) and as the QD concentration was increased (Fig. S2†). However, these apparent differences in surface morphologies were not fully confirmed by XRD analysis (Fig. S1 and Table S1†), which can probe deeper into the film and it provides a more reliable and quantitative measure of crystallinity.

Our XRD analysis shows that both the peak ratio and the full width at half maximum (FWHM) of the main MAPI diffraction peaks at 14.1° and 28.6° (Fig. S1†), corresponding to the (110) and (220) planes,^27^ did not appreciably change with different QD concentrations (Table S1†). However, differences between MAPI and MAPI–MoS_2_ films can be observed with the FWHM of the MAPI-only film exhibiting larger values, possibly indicating smaller grains.

As the film's structural integrity and crystallinity were not visibly impacted by the different QD concentrations, for the range studied here (0.2–0.6 mg mL^−1^), we selected the concentration of 0.4 mg mL^−1^ (used hereafter) on the basis of absorption characteristics, *i.e.* we selected the concentration that produced the film with the highest absorption and used this to compare directly with MAPI-only films and devices.

Fig. 1a and c shows the XRD patterns of MAPI and MAPI–MoS_2_ (0.4 mg mL^−1^) thin films. The XRD pattern of the MAPI-only film (Fig. 1a) shows the presence of a diffraction peak at 12.8° corresponding to the (001) plane of PbI_2_, which can be attributed to initial surface degradation of the film into PbI_2_.^28^ This is negligible or absent in the MAPI–MoS_2_ film (Fig. 1c), suggesting slower degradation of the composite film. In this case, films were fabricated under ambient conditions and XRD measurements were performed on the same day after the fabrication.

**Fig. 2:**
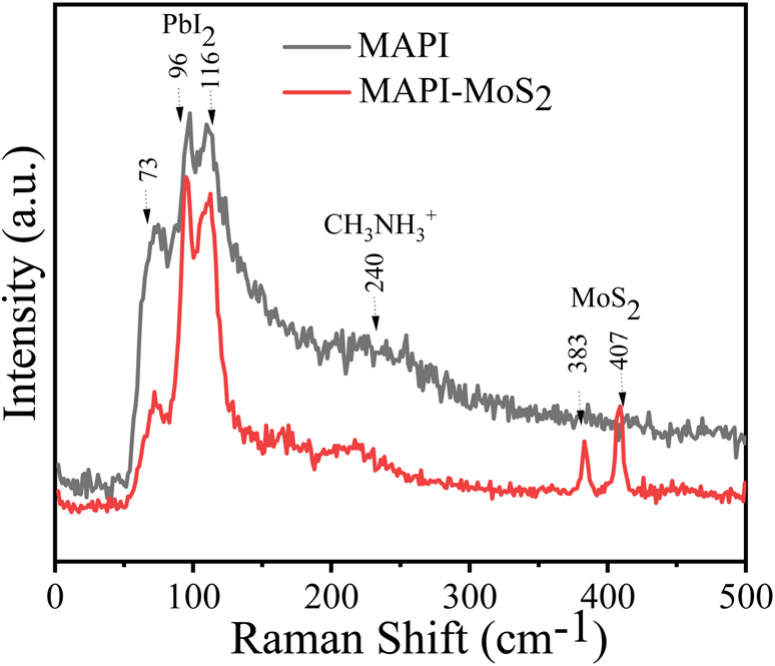
Raman spectra of MAPI and MAPI–MoS_2_ films.

Fig. 2 shows the Raman spectra of MAPI and MAPI–MoS_2_ films. Raman spectroscopy is a well-established technique to probe organic–inorganic perovskite layers and 2D transition metal dichalcogenides. To avoid unintended sample degradation and undesired heating, Raman measurements were performed using a 100-mW laser excitation power. The Raman bands in Fig. 2 at lower wavenumbers (50–300 cm^−1^) correspond to MAPI and those at higher wavenumbers (350–500 cm^−1^) correspond to MoS_2_ structures. From Raman analysis, we observed pristine perovskite vibrational bands at ∼240 cm^−1^ along with additional bands at 73 cm^−1^, 96 cm^−1^ and 116 cm^−1^ respectively, which correspond to the degradation of the perovskite structures (PbI_2_ in Fig. 2).^29^ The Raman bands at higher wavenumbers located at 383 cm^−1^ and 407 cm^−1^ correspond to the in-plane (E12g), and out-of-plane (A1g) vibrational modes of MoS_2_.^29^ This analysis shows that MoS_2_ QDs were successfully incorporated in the MAPI films. XPS analysis corroborates these results, showing typical Mo 3d and S 2 s peaks (Fig. S3†); however, sulphur generally presents a much weaker signal than molybdenum and we were unable to identify any specific bonding with the MAPI structure such as Pb–S.

**Fig. 3:**
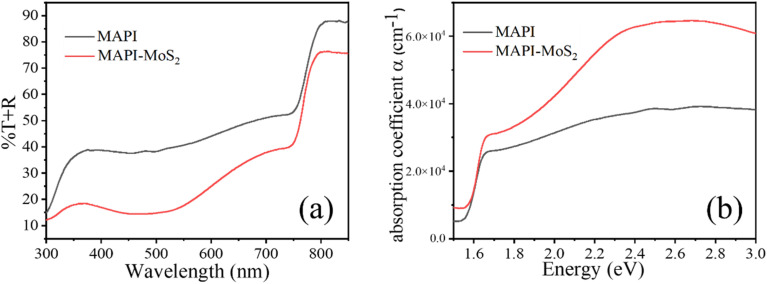
(a) UV-vis transmittance spectra of MAPI and MAPI–MoS_2_ films (0.4 mg mL^−1^); (b) absorption coefficient of MAPI and MAPI–MoS_2_ (0.4 mg mL^−1^) at different energies between 1.5 eV and 3 eV calculated from transmittance spectra.

In order to assess the opto-electronic properties of the films as relevant for photovoltaics, we first carried out UV-vis measurements, reported in Fig. 3a, which show higher absorptance for the MAPI–MoS_2_ films throughout the wavelength range compared to the MAPI film. Also, the absorptance of the MAPI–MoS_2_ films increases further in the 500–700 nm region where the absorption of MoS_2_ QDs is expected to increase (see Fig. S4 in the ESI† for MoS_2_ QD optical properties). This is clearer when the absorption coefficients are plotted, as shown in Fig. 3b where the MoS_2_ QDs contribute to a strong enhancement of the absorption coefficient above its bandgap from ∼1.8 eV, while the absorption coefficient of the MAPI film is of the same magnitude as reported in the literature.^30^

**Fig. 4:**
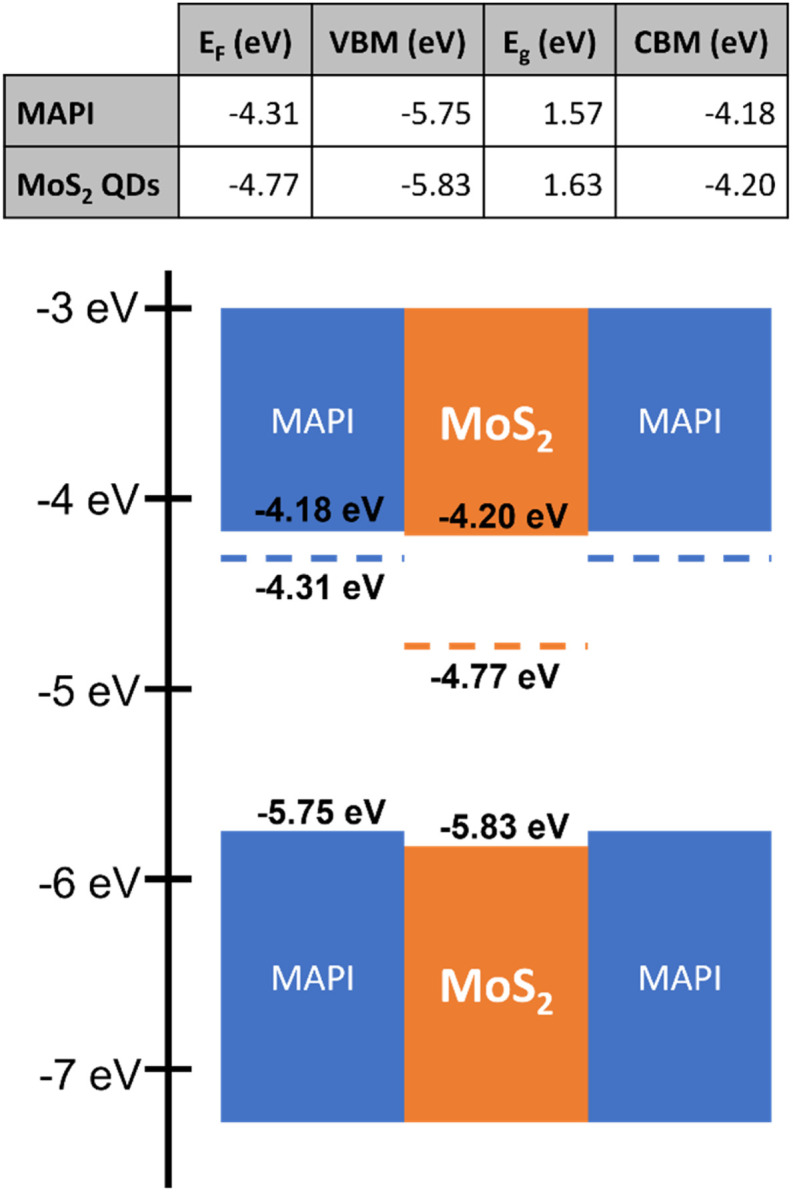
Experimental determination of the Fermi level of MAPI and MoS_2_ QDs from UPS measurements. The band gap was obtained from Tauc plots. The conduction band minimum (CBM) was determined by adding the band gap to the measured VBM. The corresponding energy diagram is also included.

We also measured the Fermi level (*E*_F_) and valence band maximum (VBM) of MAPI and MoS_2_ QDs (Fig. 4) by ultraviolet photoelectron spectroscopy (UPS) and then determined the conduction band minimum (CBM) by adding the bandgap (*E*_g_) obtained from Tauc plots of UV-vis measurements (Section-D in the ESI†).

Based on these measurements, we have depicted the band energy diagram of MAPI and MoS_2_ QDs (Fig. 4). Unfortunately, neither the non-equilibrated nor the equilibrated energy level alignment between the QDs and MAPI is ideal for carrier generation in the QDs and subsequent transfer to MAPI for transport, specifically for the holes, as energy barriers will be formed at the valence band edges. However, we expect that in the presence of a sufficiently strong electric field, when the absorber layer becomes fully depleted, carriers may still tunnel through thinned energy barriers. This generally occurs even when energy level misalignment exists between perovskite absorbers and oxide transport layers.^31–33^ These observations justify further assessments and the investigation of the impact of the QDs on the performance of full device structures.

**Fig. 5:**
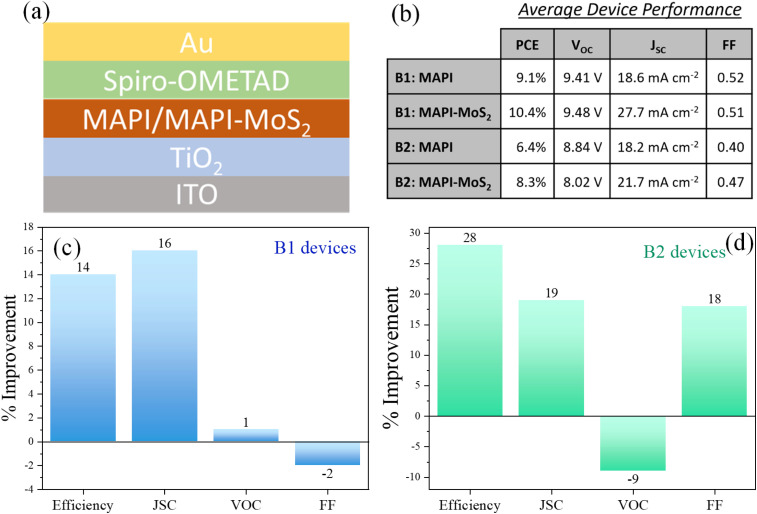
(a) Diagram of the device structure. (b) Average device performance parameters. (c and d) Changes in performance parameter of the MAPI–MoS_2_ devices with respect to the corresponding MAPI-only devices for the two sets of solar cells prepared in different laboratories (B1 and B2): power conversion efficiency (PCE), open circuit voltage (*V*_OC_), short circuit current density (*J*_SC_) and fill factor (FF).

We therefore developed device test structures to evaluate the potential application of these composite films as the absorber layer for PV devices. In our device architectures we have used TiO_2_ as the electron transport layer (ETL) and Spiro-OMETAD as the hole transport layer (HTL). The detailed procedure for device fabrication is provided in the experimental section. The structure of the control device was ITO/TiO_2_/MAPI/Spiro-OMETAD/Au. Fig. 5a depicts the complete device stack with either MAPI or MAPI–MoS_2_ sandwiched between the ETL and HTL. In order to confirm the impact of incorporating MoS_2_ QDs in the MAPI absorber, devices were fabricated in two different laboratories by two different researchers' teams, resulting in two different device batches, B1 and B2. The same fabrication procedures were followed, except that B2 devices were fabricated in a glove box while B1 devices were produced in open air with active device areas of 4 mm^2^ (B1) and 25 mm^2^ (B2). A summary of average values of the performance parameters is provided in the table of Fig. 5b, where for instance the average PCE of B1-devices increased from 9.1% to 10.4% when QDs were introduced and B2-devices also showed improvements from 6.4% to 8.3%. Average values of the open circuit voltage (*V*_OC_), short-circuit current (*J*_SC_) and fill factor (FF) for all the different types of devices are also included in the table of Fig. 5b. In addition, the *J*–*V* characteristics of the champion device and complete statistical data of all the performance parameters for both B1 and B2 devices are included in the ESI (Fig. S10–S12†). Better device performance was achieved with B1-devices (Fig. 5b), while B2 devices were likely impacted by the larger device area, which highlights that improvements in the fabrication processes are required for large area deployment.^26^Fig. 5c and d reports the relative improvements with respect to MAPI-only devices for all four performance parameters in both device batches. The introduction of MoS_2_ QDs consistently increased the overall device PCE in both B1 and B2 devices (Fig. 5c and d) with average PCE improvements of 14% (B1) and by 28% (B2).

The consistent increase in PCE directly result from relative improvements in the short circuit current density (*J*_SC_) for both B1 and B2 devices with MoS_2_ QDs (Fig. 5c and d). The increase in photocurrent can be attributed to enhanced carrier generation/collection as a result of the improved absorption coefficient (Fig. 3b) and possible MAPI defect passivation by MoS_2_ QDs. The open-circuit voltage (*V*_OC_) and fill factor (FF) exhibited smaller and mixed results, with a negative change of up to 9% in *V*_OC_ for B2-devices and only a 4% change in FF for B1-devices, with negligible impact on the *J*_SC_ and PCE improvements.

**Fig. 6:**
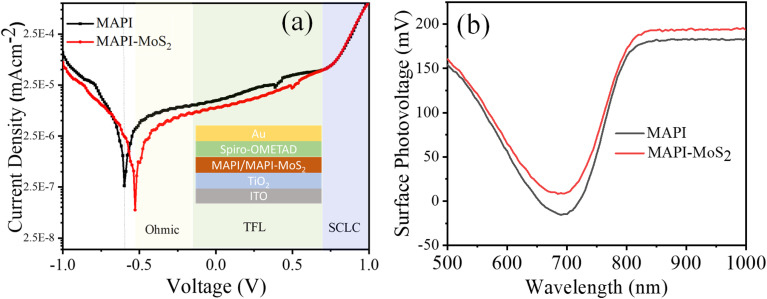
(a) Typical dark current and (b) surface photovoltage spectroscopy of MAPI and MAPI–MoS_2_ devices from batch B1.

In order to understand better the impact of MoS_2_ QDs, we carried out further measurements on the B1 devices. Representative dark-current plots of MAPI-only and MAPI–MoS_2_ devices are shown in Fig. 6a, showing lower dark current in the composite film.

The increase in dark current is caused by the migration of charge carriers due to the presence of defects, thus further supporting the presence of lower defect density in the composite film. The decrease in leakage current density also supports the passivation effect of MoS_2_. We also measured the surface photovoltage spectra of representative MAPI and MAPI–MoS_2_ films in the wavelength range of 500 nm to 1000 nm (Fig. 6b, B1-devices). The lower MAPI–MoS_2_ signal is indicative of better charge separation capacity compared to the MAPI-only film.^34,35^

To further assess the passivation effect of MoS_2_ QDs on the active layer and corresponding charge carrier recombination, electrochemical impedance spectroscopy (EIS) was performed (Fig. S9 in the ESI†). The obtained EIS spectra were best fitted using a previously reported model, considering a steady state concentration of the mobile carrier.^36^ Compared to the MAPI-only device, higher recombination resistance was observed for the MAPI–MoS_2_ device, indicating reduced charge carrier recombination losses due to the presence of MoS_2_ QDs in the active layer.^19,37^ The measurements of dark current together with surface photovoltage spectroscopy and electrochemical impedance spectroscopy provide independent and consistent evidence that the introduction of MoS_2_ QDs effectively passivates defects and enhances transport. These results are consistent with the higher PCE and short-circuit current density of the MAPI-MoS_2_ devices.

Overall MoS_2_ QDs contribute to enhanced light absorption as evidenced by the measured absorption coefficient shown in Fig. 3. This is directly reflected in an increased short-circuit current density and higher PCE. While the capture cross section of the MAPI interface defects is not expected to change, a reduced defect density, as suggested by dark current measurements (Fig. 6a), surface photovoltage spectroscopy (Fig. 6b) and electrochemical impedance spectroscopy (Fig. S9†), may have positively impacted the collection efficiency, thus contributing to higher device performance with QDs.

Carriers generated in the bulk MAPI layer are expected to dissociate at the interface with the corresponding transport layers. However, in this device architecture, the dissociation and transport of carriers generated within the QDs present a far more complex scenario. Carrier dissociation may occur at the MAPI–QD interface, for instance, facilitated by the electric field and by the electron affinity of the MoS_2_ QDs, which is more negative with respect to MAPI (Fig. 4). However, we should note that carriers may not dissociate at this interface and that excitons instead, *i.e.* carriers in their bound state, may transfer from the QDs to the MAPI, with dissociation occurring within the MAPI under the electric field or at the MAPI–transport layer interface.

Our results have therefore shown that while the device performance is improved, the energy level alignment is not ideal as it creates barriers that limit the collection efficiency of holes generated within the QDs or the transfer of excitons. Improvements in energy level alignment, which avoid the formation of barriers, could help harnessing the benefits of perovskite–QD composite layers, where for instance a type-I alignment should be favoured.^21^ This could be achieved for instance through appropriate material selection as well as by different forms of QD doping or surface engineering.^38,39^

With respect to device and film fabrication, it should be noted that the ionic liquid assisted grinding synthesis of MoS_2_ QDs is scalable and well-suited for the realization of large-area devices. In addition, the introduction of MoS_2_ QDs in the fabrication process does not require substantial additional steps and does not impact the scalability of the current perovskite solar cell fabrication methodologies. The chemical composition of MoS_2_ QDs also ensures that the environmental impact is limited and does not exacerbate existing challenges in deploying perovskite photovoltaics.

## Conclusion

We have successfully incorporated MoS_2_ QDs into MAPI and highlighted the possibility of charge separation and collection with this configuration of carriers generated from the QDs. The alignment between the MAPI layer and MoS_2_ QDs can be further optimized to avoid the formation of energy barriers for carrier transport; however both passivation of MAPI defects and increased absorption have contributed to an overall increase in device efficiency. These results have been demonstrated in separate laboratories, confirming the consistency of the contribution from the QDs. The use of composite films integrating QDs is very promising not only for the specific MAPI–MoS_2_ pair but also for other materials that enable suitable band energy alignment, potentially paving the way toward high efficiency QDs solar cells.

## Conflicts of interest

There are no conflicts to declare.

## Supplementary Material

NA-OLF-D5NA00485C-s001

## Data Availability

This paper is accompanied by representative samples of experimental data and the relevant numerical tabulated raw data are available from the University of Strathclyde's Research Portal at https://doi.org/10.15129/a4aad5a4-1052-42bb-a31c-60fd167fd9b2. Detailed procedures explaining how these representative samples were selected, and how these experiments can be repeated, are provided in the corresponding sections of this paper. Additional results and raw data underlying this work are available in the ESI† or on request, following instructions provided at https://doi.org/10.15129/a4aad5a4-1052-42bb-a31c-60fd167fd9b2.
