# Supplementary material for: MAPI–MoS2 quantum dot composite films as active layers for efficient photovoltaics
Source: Nanoscale Adv. 2025 Jun 23;7(15):4628–35. doi: 10.1039/d5na00485c (PMC12183647; doi:10.1039/d5na00485c)
Supplement: NA-007-D5NA00485C-s001 [file NA-007-D5NA00485C-s001.pdf]

Supplementary Information (SI) for Nanoscale Advances.
This journal is © The Royal Society of Chemistry 2025

2MAPI-MoS_2 quantum dots composite film as active layer for efficient photovoltaics

Subha Sadhu,[1] Ankur Kambley,[2] Talitha RC Santos,[3] Abhijit Ganguly,[4] Slavia Deeksha Dsouza,[4] Dilli Babu Padmanavan,[4] Pagona Papakonstantinou,[4] Paul Maguire,[4] Vladimir Svrcek,[2] Davide Mariotti[5]

[1]Department of Chemistry, Institute of Science, Banaras Hindu University, Varanasi, India

[2]Renewable Energy Research Center, National Institute of Advanced Industrial Science and Technology (AIST), Tsukuba, Japan

[3]Group of Nanostructured Devices, Federal University of Paraná (Physics Department), Curitiba, PR, Brazil

[4]School of Engineering, Ulster University, Belfast, UK

[5]Department of Design, Manufacturing & Engineering Management, University of Strathclyde, Glasgow, UK

Section A. Measurements on the films with other MoS_2 QDs concentrations

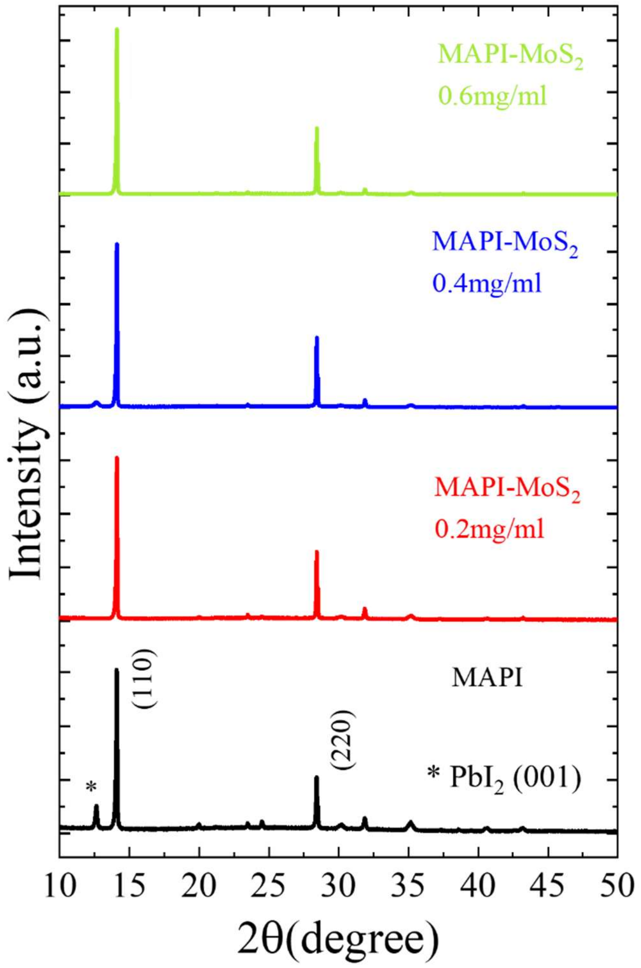

Figure S1. XRD patterns of MAPI-MoS_2 film with different amount of MoS_2 QDs.

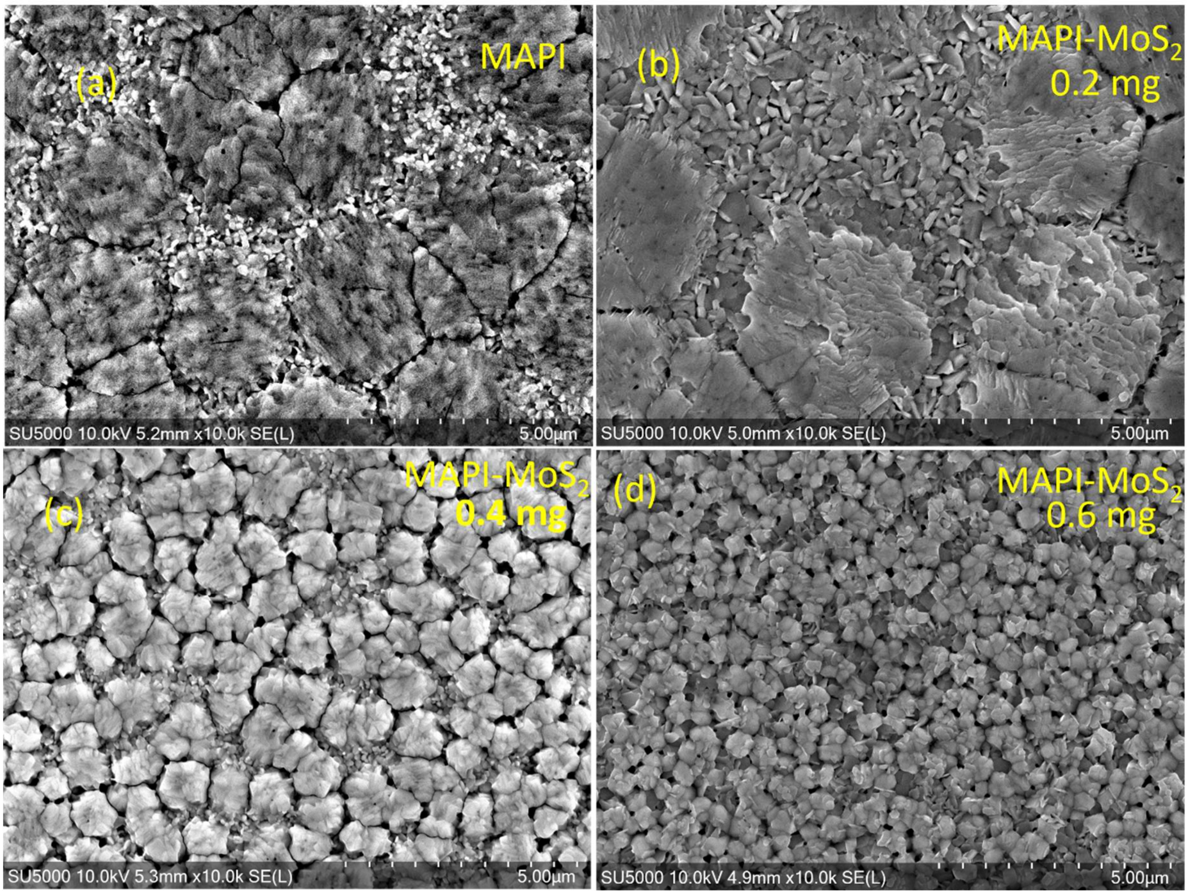

Figure S2. SEM images of the films with and without QDs at different concentrations.

Table S1. Peak ratios and FWHM calculated from XRD spectra at different QDs concentrations

QD concentration / mg mL-1	Peak ratio (110)/(220)	FWHM @ 14.1° (110)	FWHM @ 28.6° (220)
0.0	2.91	0.1149	0.1414
0.2	2.83	0.0927	0.1146
0.4	2.81	0.0901	0.1142
0.6	2.82	0.0923	0.1165

Section B. Further film characterization

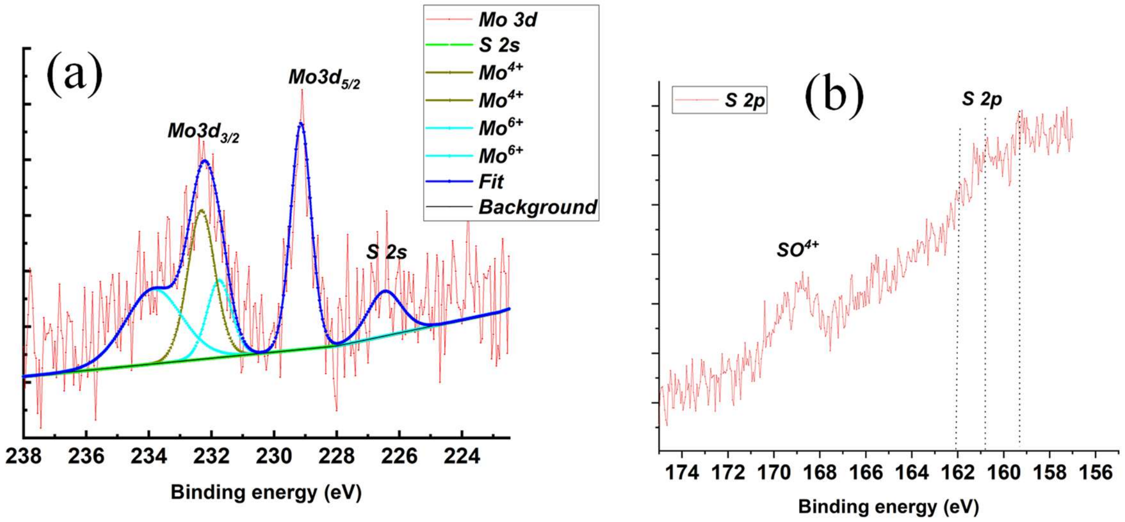

Figure S3. XPS spectra of MAPI-MoS_2 thin film with 0.4mg/mL concentration showing the presence of Mo (a) and S (b).

Section C. MoS_2 QDs optical properties

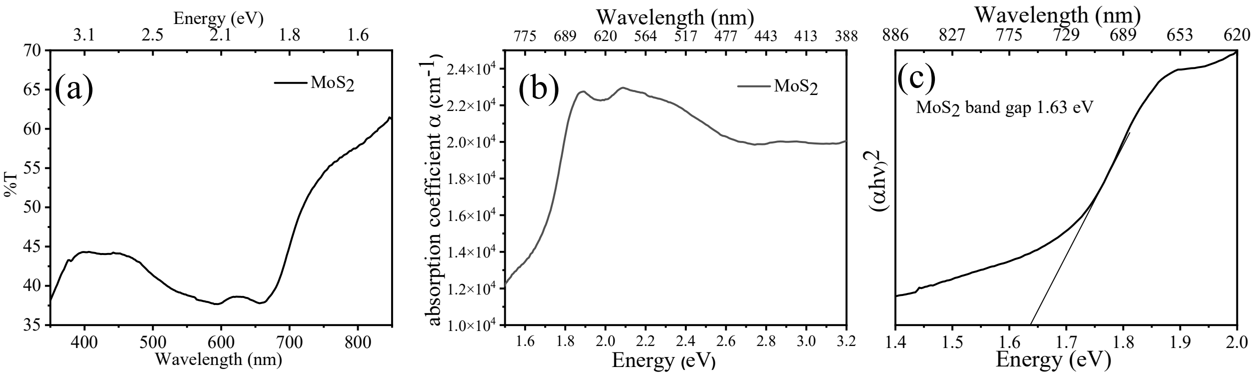

Figure S4. UV-Vis transmittance spectra (a), absorption coefficient (b) and Tauc plot (c) of MoS_2. Absorption coefficients were measured from the transmission spectra (T) considering negligible reflection. Absorption coefficient is equal to -ln(T)/d, where d is the thickness of the film and T is the transmittance. The thickness of the film is ~ 500 nm which is measured from profilometer.

Section D. Measurements of the energy band levels

Table S2. Energy levels of MAPI, MAPI-MoS_2 and MoS_2 QDs determined by APS and UPS measurement. Bandgap values are from Tauc plot.

	E_F (eV) UPS	VBM (eV) APS	VBM (eV) UPS	E_g (eV) UV-Vis	CBM (eV) $VBM + E_g$
**MAPI**	-4.31	-5.6	-5.75	1.57	-4.18
**MoS_2 QDs**	-4.77	-5.7	-5.83	1.63	-4.20

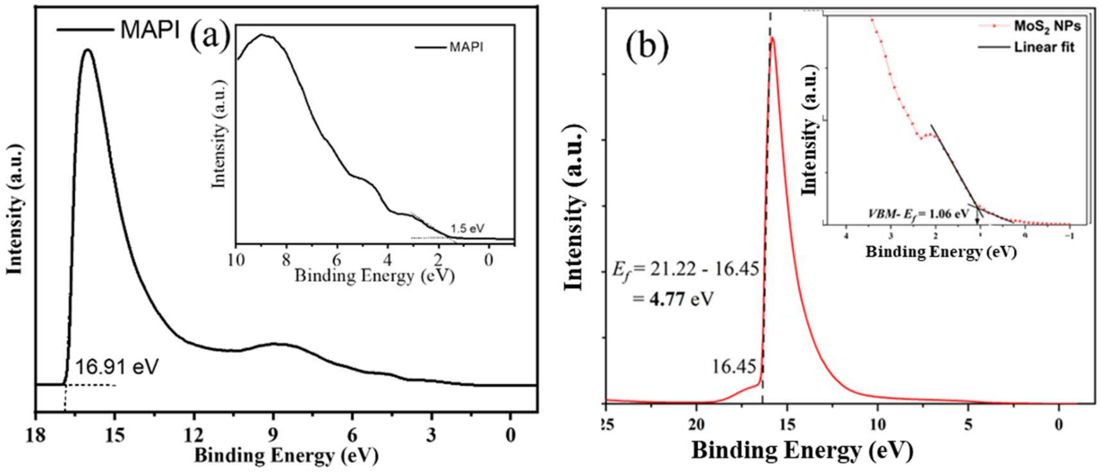

Figure S5. UPS spectra of (a) MAPI and (b) MoS_2 thin film showing cut off value and onset region. Insets show the zoom view of the onset region. (b) is reproduced with permission.

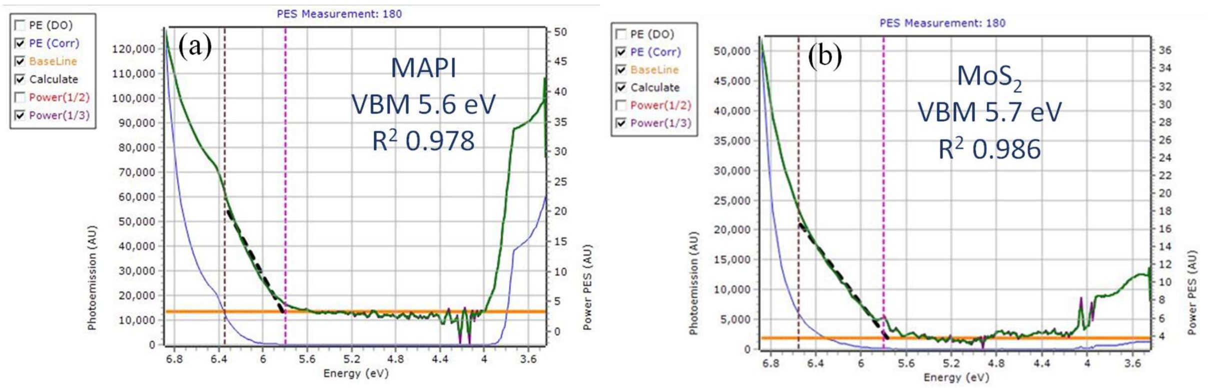

Figure S6. APS spectra of (a) MAPI, and (b) MoS_2.

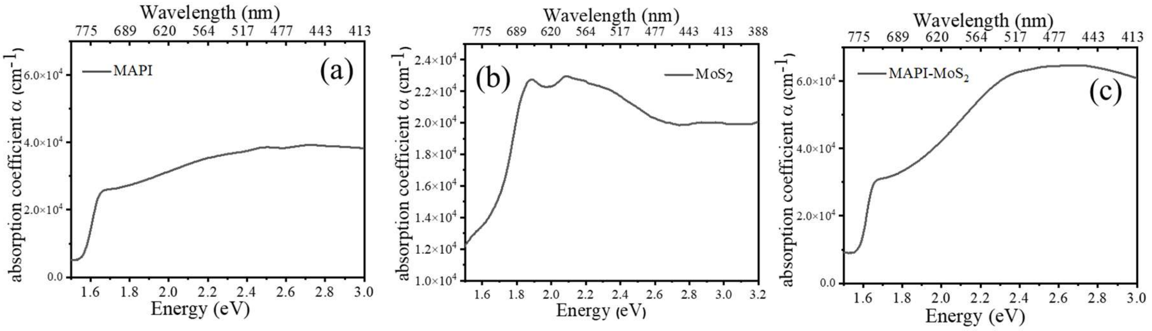

Figure S7. Absorption coefficient of (a) MAPI, (b) MoS_2 and (c) MAPI-MoS_2 thin film. Absorption coefficients were measured from the transmission spectra (T) considering negligible or similar reflection in all three films. Absorption coefficient is equal to -ln(T)/d, where d is the thickness of the film and T is the transmittance. The average thickness of the MAPI and MAPI-MoS_2 is ~180-300 nm. The thickness of the film was measured by profilometer and cross-sectional SEM and resulted to be 300, 500 and 350 nm for MAPI, MoS_2 and MAPI-MoS_2 respectively.

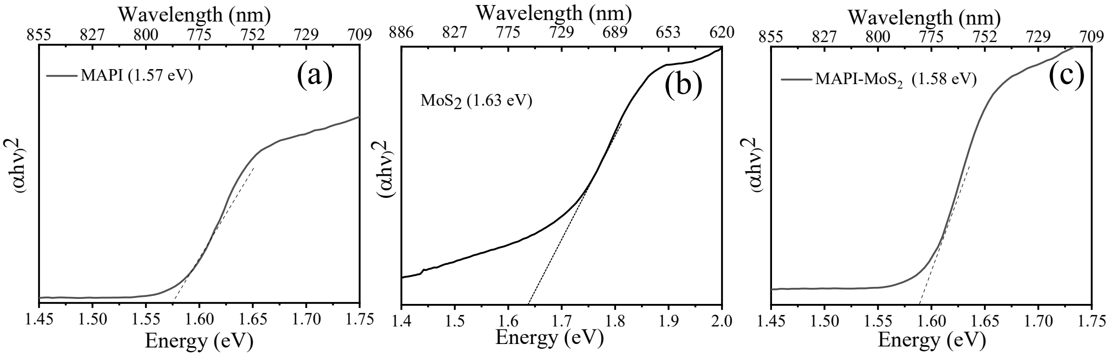

Figure S8. Tauc plot of (a) MAPI, (b) MoS_2 and (c) MAPI-MoS_2 thin film. Direct bandgap was used; hence the band edge energy of the thin films is calculated by plotting $(\alpha h\nu)^2$ against hν (where a is absorption coefficient and hν is photon energy). Band gap of the thin film is calculated by extrapolation of Tauc plot.

Section E. Further device measurement and statistics

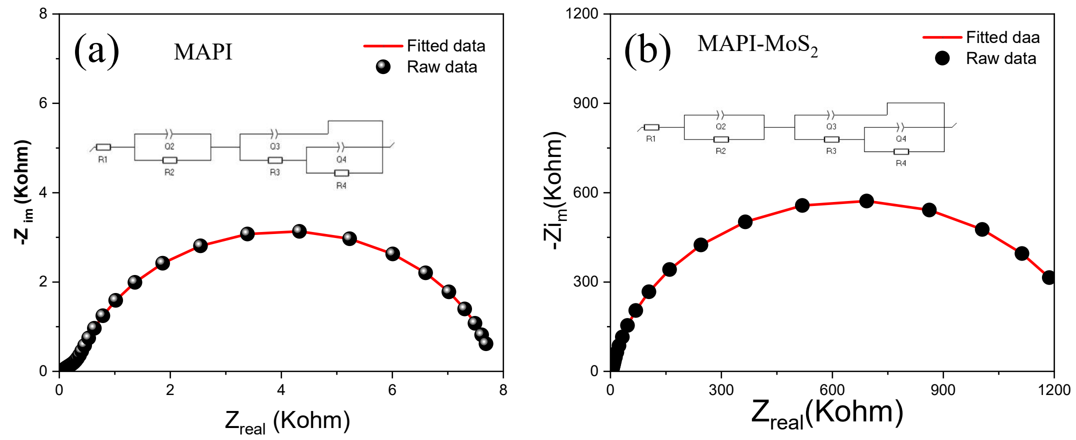

Figure S9. Nyquist plot of device in dark using (a) MAPI and (b) MAPI-MoS_2 as active layer. Inset shows the circuit model used for fitting.

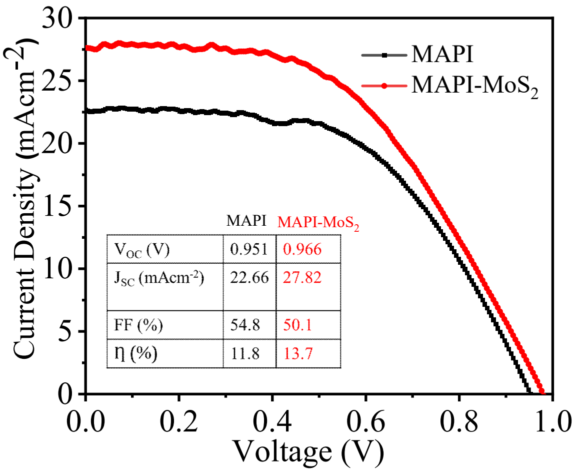

Figure S10. J-V characteristic of the champion device from B1 devices.

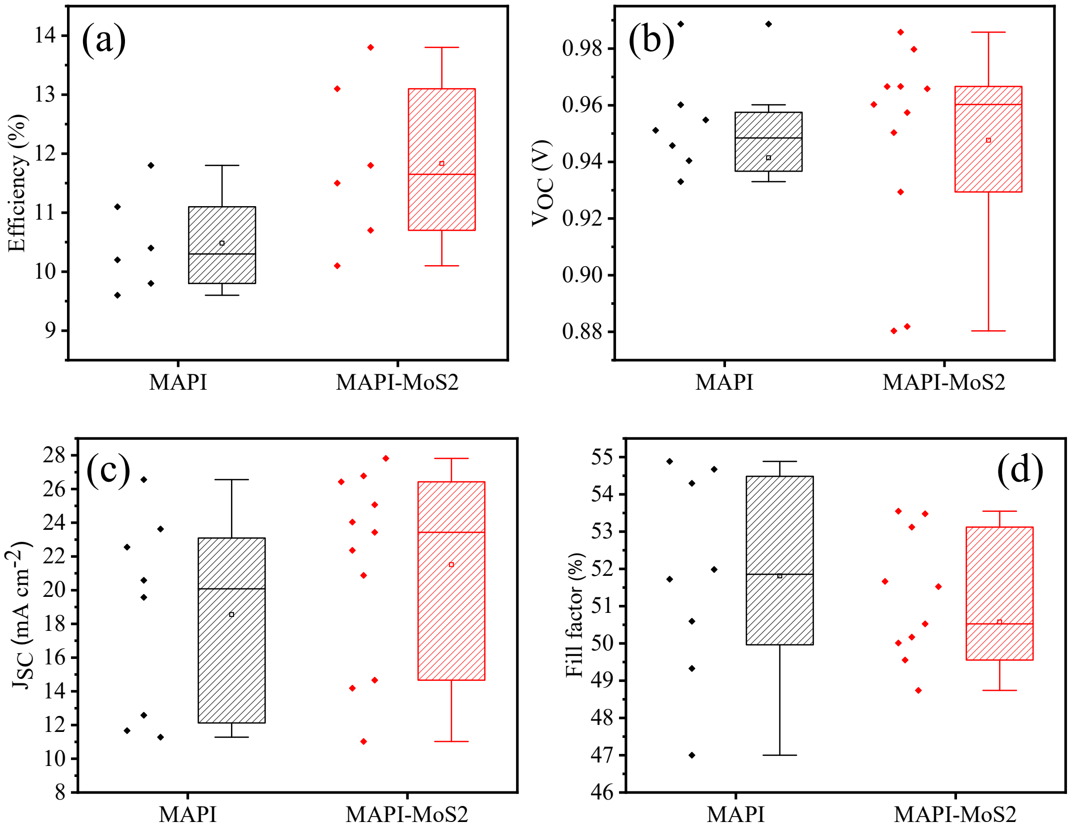

Figure S11. (a-d) Statistics of device performance parameters for MAPI and MAPI-MoS_2 devices, batch B1: efficiency, open circuit voltage (V_{OC}), short circuit current (J_{SC}) and fill factor (FF). Reverse measurements and devices at the center of the substrates were here considered. Excluded were also a few devices that did not work (i.e. did not produce a clear photocurrent, 5 out of 53).

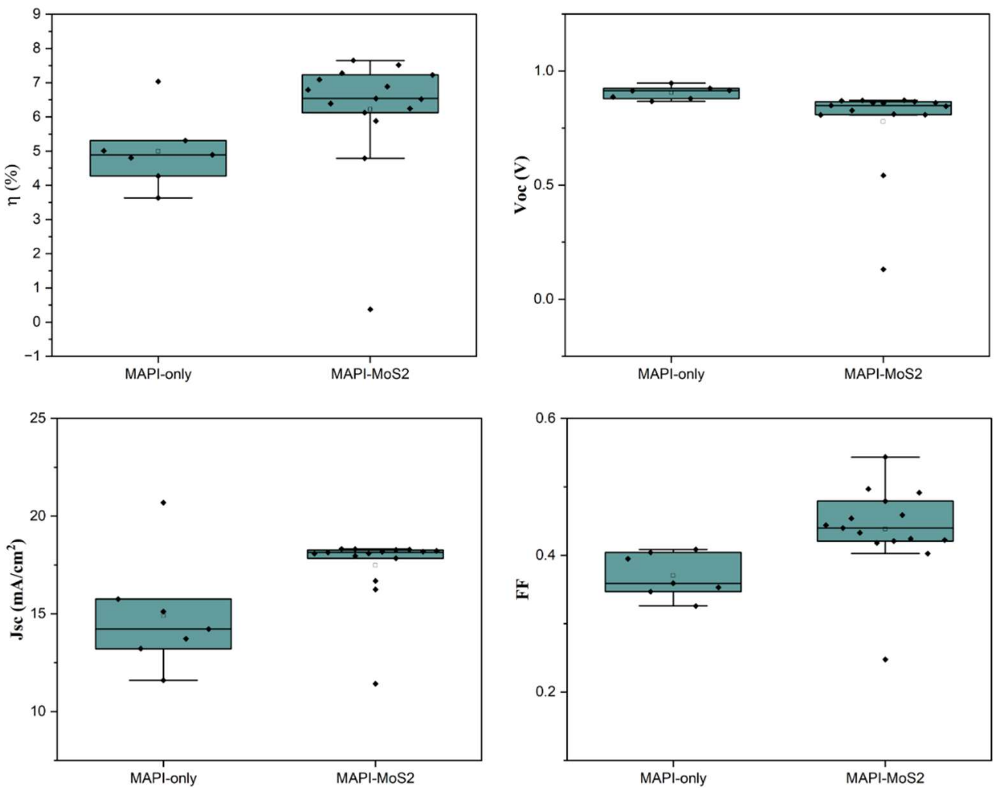

Figure S12. (a-d) Statistics of device performance parameters for MAPI and MAPI-MoS_2 devices, batch B2: efficiency, open circuit voltage (V_{OC}), short circuit current (J_{SC}) and fill factor (FF).
